# Algorithm for dermocosmetic use in the management of cutaneous side-effects associated with targeted therapy in oncology

**DOI:** 10.1111/jdv.12082

**Published:** 2013-02-01

**Authors:** B Dreno, RJ Bensadoun, P Humbert, J Krutmann, T Luger, R Triller, A Rougier, S Seité

**Affiliations:** 1 Department of Cancero-Dermatology, Hôtel Dieu, CHU NantesFrance; 2 Department of Radiological Oncology, CHU PoitiersFrance; 3 Department of Dermatology, Research and Studies Center on the Integument (CERT), Clinical Investigation Center (CIC BT506), Besançon University Hospital, University of Franche-Comté, INSERM UMR1098Besançon, France; 4 IUF-Leibniz Research Institute for Environmental Medicine, Heinrich-Heine-UniversityDüsseldorf, Germany; 5 Department of Dermatology, University of MünsterGermany; 6 Centre of Dermatology, Hertford British HospitalLevallois-Perret, France; 7 La Roche-Posay Laboratoire PharmaceutiqueAsnières, France

## Abstract

Currently, numerous patients who receive targeted chemotherapy for cancer suffer from disabling skin reactions due to cutaneous toxicity, which is a significant problem for an increasing number of patients and their treating physicians. In addition, using inappropriate personal hygiene products often worsens these otherwise manageable side-effects. Cosmetic products for personal hygiene and lesion camouflage are part of a patients’ well-being and an increasing number of physicians feel that they do not have adequate information to provide effective advice on concomitant cosmetic therapy. Although ample information is available in the literature on pharmaceutical treatment for cutaneous side-effects of chemotherapy, little is available for the concomitant use of dermatological skin-care products with medical treatments. The objective of this consensus study is to provide an algorithm for the appropriate use of dermatological cosmetics in the management of cutaneous toxicities associated with targeted chemotherapy such as epidermal growth factor receptor inhibitors and other monoclonal antibodies. These guidelines were developed by a French and German expert group of dermatologists and an oncologist for oncologists and primary care physicians who manage oncology patients. The information in this report is based on published data and the expert group’s opinion. Due to the current lack of clinical evidence, only a review of published recommendations including suggestions for concomitant cosmetic use was conducted.

## Introduction

Targeted chemotherapy is associated with cutaneous side-effects, which is becoming more and more a problem for an increasing number of patients and their treating physicians. However, with appropriate skin care, in association with pharmaceutical treatment, these reactions can be adequately managed.

In recent years, the improved survival outcome and the superior safety profile of targeted molecules in chemotherapy have revolutionized the treatment of haematological malignancies and solid tumours of head and neck, breast, lung, liver, kidney or colorectal origin and more recently, melanoma.^1^–^4^ Despite the improved systemic tolerability towards chemotherapeutic agents, they are nevertheless associated with adverse cutaneous reactions. If not managed appropriately, these reactions can become uncomfortable and disfiguring.^5^–^12^ Although not life threatening, they are becoming an increasingly important preoccupation for both patients and their treating physicians as patient survival rates improve. Skin, ocular, nail and hair toxicity have been comprehensively described in the literature as common side-effects to expect.^5^–^15^ Typical reactions include folliculitis (skin rash), xerosis, pruritus, hand and foot erythema and an increased sensitivity to ultraviolet radiation. Symptoms usually appear early in treatment and although usually mild at onset, can become severe and maybe considered as a class effect.^16^ They can lead to serious morbidities that impair quality of life.^17^–^18^ As symptoms are dose dependent and considered a validated measure for efficacy, patients are encouraged to manage adverse cutaneous reactions as a part of their treatment.^3^–^20^

Conversely, one recent retrospective survey of oncologists showed an alarming number of dose reductions and treatment discontinuations due to skin rash.^13^ Such dose reductions may be considered as potentially detrimental to the treatment outcome. Consequently, appropriate management of dermatological toxicity is an important issue throughout treatment.

The primary role of skin-care products is to provide exogenous support that maintains the epidermal barrier intact.^21^ Skin hydration relieves symptoms associated with dry skin and reduces further aggravation associated with pruritus and leading to secondary infections.^22^–^23^ In addition to their role of barrier function maintenance, skin-care products are intended for cleaning the skin. As personal hygiene is part of most cultures today, patients do need advising on appropriate skin care.^24^ Although many products are appropriate, a certain number hygiene products are not, as they may aggravate symptoms.^9^–^24^ Some dermatological skin-care products are formulated for and tested on fragile, pathological and sensitive skin. Such products may be considered as more appropriate for concomitant management of cutaneous side-effects.^9^

With an increasing overall survival, primary care providers are playing an increasingly important role in managing oncology patients, and may therefore need some guidance in managing adverse cutaneous reactions.^25^

Therefore, the objective of this study is to provide oncologists and primary care physicians managing cancer patients with a therapeutic algorithm based on an extensive literature review of cosmetics associated with targeted therapy for cancer as well as the expert’s opinion based on their experience with the use of dermatological skin-care products and cosmetics.

## Methodology

The present recommendations focusing on skin, mucosa and nail disorders following oncology treatments were developed following proposals and conclusions reached during a consensus meeting held in November 2011. The working group consisted of six independent European dermatologists and one oncologist.

Prior to the meeting, an *ad hoc* literature review (using Pubmed and BIOSIS) was performed. The key words chosen were emollient + cancer + skin, sunscreen + cancer + skin, hygiene + cancer, make-up + cancer.

During the meeting, the literature concerning different cutaneous toxicities related to targeted therapy and chemotherapy as well as to quality of life was reviewed. The working group discussed appropriate dermatological products for each cutaneous symptom according to the available literature, and completed their recommendations with current practices in France and Germany and personal experience.

Dermatological skin care was defined as cleansing, moisturizing, personal hygiene and photoprotection using products having a good tolerance profile, tested on pathological skin.

There are different classifications for the degree of skin toxicity. However, the working group referred to The National Cancer Institute Common Toxicity Criteria (CTCAE) version 4, which is a widely known and accepted scale for the assessment of adverse events.^26^ This scale provides objective criteria that reflect the current management of cutaneous toxicities associated with targeted therapy.

## Literature review

Evidence-based support for the use of dermatological cosmetics and make-up as adjunctive therapy remains scarce. Practice is based on anecdotal reports or studies with limited control. Table 1 provides an overview of key studies conducted recommending the use of cosmetics as part of side-effect management.

**Table 1:** List of key references recommending the use of cosmetic agents in combination with chemotherapy

Author	Study design	Title	Comments
Lacouture^9^	Review	Mechanisms of cutaneous toxicity	
Segaert S *et al*.^7^	Review article	Clinical signs, pathophysiology and management skin toxicity	Adequate sun protection
			Avoid skin drying cosmetic products
			An emollient on hands and limbs to prevent fissures
Segaert S *et al*.^6^	Review article	Skin toxicities of targeted therapy	Maximize skin hydration
			Sun protection
		Rash: Avoid retinoids	
			Xerosis: oil-in-water creams, 5-10% urea
			Paronychia: Topical antiseptic
Robert C *et al*.^5^	Review article	Cutaneous side-effects of kinase inhibitors and blocking antibodies	Camouflage cosmetics for *folliculitis*
			Xerosis: prescribe 5–10% urea
Ouwerkerk, J *et al*.^30^	Review article	Anti EGFR for metastatic colorectal cancer	Sunscreen >15+
			Avoid cleaning detergents
			Mild body cleansers
			Moisturizers
			Avoid alcohol-based products
			Cosmetics to conceal rash
		Gentle non-alcohol-based cleansers	
Perez-Soler *et al*.^14^	Guideline	HER1/EGFRI assoc rash: future directions for mgt and outcomes from the HER1/EGFRI rash management forum	Cover rash with make-up
			Use a skin-friendly make-up remover
			Use emollients to prevent skin dryness
			Use a good sunscreen
			Avoid over-the-counter acne medication
Burtness *et al*.^1^	Guideline	Task force report. Management of dermatological and other toxicities associated with EGFRI in patients with cancer.	Initiate treatment early
			Avoid using antiacne medication
			Thick emollients
			Mild soap
Bernier *et al*.^36^	Guideline	Consensus guidelines for the mgt of radiation dermatitis and acne-like rash in patients receiving radiotherapy+ EGFRI for the treatment of head and neck squamous cell carcinoma	Use gentle cleansers
			Topical cosmetics for symptomatic relief
			Avoid sun exposure (mineral sunblocks or clothing)
			Avoid perfumes and alcohol-based lotions.
Lynch TJ *et al*.^6^	Guideline	EGFRI dermatologic toxicity overview of outcomes.	Expert opinion
			Initiation of treatment, moisturize dry areas twice daily
			Thick alcohol-free emollient
			Broad-spectrum sunscreen 15+ or higher
			Add Medical treatment if severity increases.
Infections			
Grenader T *et al*^23^	Case report	Staph aureus on culture following erlotinib treatment			

Author	Study Design	Population	Intervention	Sample size	Outcome measurement	Results	
Fluhr 21	3-week, controlled, monocentric, study	Experiencing dry, sensitive skin during chemotherapy	Application of acidic pH 5.5 washing emulsion and body lotion	30	Skin-surface pH	Improved skin physiology
					TEWL	Sig increase in stratum corneum hydration *P* < 0.001
					Corneometer	Reduced TEWL *P* > 0.007
					Sebumeter	Significant reduction of skin symptoms *P* < 0.001
					Clinical evaluation
Roé E *et al*^29^	Prospective, uncontrolled study of toxicity	Cetuximab or erlotinib	Severity graded	30	Application of an emollient for treatment of xerosis	Good control of symptoms.	No description of which cosmetic products used when.
					Antiseptic soaps
Pre-emptive skin-care management reduces incidence and severity of skin rash							
Wohlrab^34^	Monocentre POC	Breast cancer chemotherapy	Application of an emollient	13	DLQI – quality-of-life questionnaire	Maintains quality of life and reduces frequency of adverse events	Poster
Ocvirk J *et al* 22	Prospective, monocentre, uncontrolled study	cetuximab Review of local practice for management of skin toxicity	Application of an emollient after the first documented cutaneous toxicity	31	Skin toxicity Classification using NCI CTCAE v3.0	29.03% grade 1 – advised emollients	Recommend prompt application of topical emollients. Add topical of systemic medication for increasing severity.
		Xerosis	Grade 1 were treated with emollients			51.61% grade 2 – advised emollients + topical antibiotic
		fissures	Grade 2 emollients+ antibiotics			19.36% grade 3 – interruption of treatment then emollients + topical antibiotics
			Grade 3 emollients+ systemic antibiotics			Xerosis – advised emollients 2–5% urea
						Fissures – creams + dexpanthenol
Use of beauty care and cosmetics improves quality of life and management of side-effects							
Boone SL *et al*^13^	Qualitative Survey	Oncology providers	51 item, open-ended questionnaire pertaining to incidence of rash among patients on EGFRI, treatment practices, patient perceptions, outcome treating rash	110		71–90% of patients experience rash.
						8% of providers surveyed obtained a dermatology consult.
						Grade 3 and 4 rash interfered with EGFRI therapy (3%).
Taggart L *et al*^39^	Pilot study	Cancer patients	Participation at a look-good–feel-better workshop	20	Self-image NSC	Improved self-image (*P* < 0.005)
					Anxiety STAI	Reduced anxiety (*P* < 0.01)
Haley *et al*^18^	Monocentre, uncontrolled study	Cancer patients receiving either cytotoxic chemotherapy, targeted, or hormonal and/or radiotherapy	Applied three test products after first toxicity visit skin and face moisturizer and face wash	99	Q of L: Skindex questionnaire	Significant decrease (*P* = 0.0003) from baseline in mean overall skindex score							
Merial-Kieny C *et al*^52^	Multicentre qualitative survey	Visible cutaneous side-effects following chemotherapy	Participated in a make-up training seminar	90	Self-completed questionnaire on tolerance, quality of life and the make-up techniques	95,4% very or satisfied with tolerance							
			Applied a hypoallergenic make-up			81,2% improved their quality of life.
Amiel P *et al*^40^	Multicentre, qualitative survey	Cancer patients	Survey of patient experience receiving various beauty-care services	60	Observation and semistructured interviews	18/40 appreciated information on treating skin problems							
						23/40 appreciated information on make-up techniques
Titeca G *et al*^41^	Prospective, randomized, controlled multicentre study	Breast cancer Antitumor chemotherapy	2H cosmetic care interview	27	VQ dermatological questionnaire	less discouraged (*P* = 0.032)							
						more self-confident (*P* = 0.032)
Williams S *et al*^46^	Outpatient chemotherapy clinics	First chemotherapy Breast cancer patients	Informational audiotapes for self care	70	STAI	Treatment group increased use of management techniques							
						Symptoms decreased

TEWL, Trans Epidermal Water Loss; NCI-CTC, National Cancer Institute cutaneous toxicity Criteria.

**Table 2:** Spectrum of dermatological reactions to EGFR inhibitors^39^

Adverse Event	Description	Frequency	Time Course
Rash (follicular-pustular)	Monomorphous erythematous maculopapular, follicular or pustular lesions, which may be associated with mild pruritis	60–80%	**Onset:** treatment week 1.
			Maximum: treatment week 3–5.
			**Resolution:** within 4 weeks of treatment cessation, may wax and wane or resolve spontaneously
Paronychia and fissuring	Painful periungual granualtion-type or friable pyogenic granuloma-like changes, associated with erythema, swelling and fissuring of lateral nailfolds and/or distal finger tufts.	6–12%	Onset: treatment month 2–4
Dry skin	Diffuse fine scaling	4–35%	Occurs after appearance of rash

## Side-effects associated with targeted chemotherapeutic agents

Cutaneous toxicity with chemotherapeutic agents is common. Although designed to target specific molecular tumour growth factors, they also target growth factors in the skin and its appendages.^6^–^9^ To date, the exact mechanisms involved in the development of cutaneous symptoms are only partly understood.^16^ However, the molecular, histological and clinical observations suggest that targeted therapies ultimately disturb skin barrier function.^9^ Clinical symptoms include disruption of the pilosebaceous follicle causing folliculitis (skin rash), alteration of the skin barrier with xerosis, cracked skin and pruritus (itchy skin). Other common reactions include hand and foot erythema, increased sensitivity to ultraviolet radiation, hyperpigmentation and finally, alteration of phaneres with paronychia.^7^–^15^ In addition to disturbed epidermal barrier function, the skin is more sensitive to allergens and open to infection.^7^,^15^

### Rash (folliculitis)

The most common reaction reported is skin rash,^1^ which appears in 43–85% of patients treated with epidermal growth factor receptor inhibitors (EGFRI)s.^5^ This rash follows a typical, chronological pattern that peaks in severity during the first 1–2 weeks.^6^–^7^ Although it is not associated with death, reports of serious morbidity have been identified.^27^ In the current absence of consistent clinical trials, patients are therefore advised to use mild skin care and photoprotection.^16^–^28^

Grade 1 rash was successfully managed with emollients and adapted skin cleansers in a local practice review.^22^ In a similar observational study, Grade 1 rash was also shown to be managed with topical antibiotics and antiseptic soap.^29^

Non-occlusive make-up with a high pigment concentration to adequately cover scars and lesions has been repeatedly suggested to cover grade 1 and 2 rash. 5,8 Furthermore, appropriate skin care and corrective make-up were shown to be tolerated by patients receiving chemotherapy in one multicentre study, and avoiding allergenic over-the-counter products is recommended.^6^–^14^ Make-up should be removed with a dermatologist-approved, low-irritant, non-alcoholic hypoallergenic remover.^14^,^30^ Over-the-counter acne products have been repeatedly contraindicated, including products containing benzoyl peroxide and topical retinoids such as tretinoin, adapalene or tazarotene. These agents generally are considered as drying the skin and causing sensations of burning, stinging and irritation, while not having shown clinical benefit in the treatment of rashes.^1^,^8^

A number of authors have discussed the growing evidence for rash severity as a surrogate marker of efficacy with certain products.^19^ Although further evidence is required to quantify these observations, authors advise continuing epidermal growth factor receptor (EGFR) inhibitor therapy in association with appropriate psychosocial support.

### Xerosis

Xerosis appears several weeks after the first EGFR treatment in up to 35% of patients.^28^–^32^ The review articles examined unanimously recommended applying emollients to ensure maximal skin hydration.^7^,^28^, Some authors found emollients containing 5–10% urea useful, while a recent monocentric proof-of-concept study suggested that the supportive application of an emollient containing niacinamide maintains quality of life and reduces the frequency of adverse events.^34^ Three international expert groups support the general use of emollients for dry skin despite the lack of prospective data.^1^,^8^ *Segaert* presents his clinical experience of switching topical treatments to oil-in-water formulations and for limbs, water-in-oil formulations for moderate-to-severe xerosis on the first sign of dryness.^7^

One single-centre, controlled, assessment of skin function in chemotherapy patients showed a significant increase in the *stratum corneum* hydration (*P* < 0.001) and a decrease in transepidermal water loss (*P *< 0.03) following prophylactic treatment with an acidic (pH 5.5) skin-care system (emollient and cleanser).^21^ *Roé et al*., in an uncontrolled trial of 30 patients also reported that moisturizers were a useful treatment for xerosis.^29^

Nursing reviews recommend proactive management of rash and xerosis.^18^–^30^ Consensus articles state treating fissures with liquid bandages or thick emollients containing 5–10% urea,^5^,^35^ _ENREF_43 and the use of antiseptic cream to prevent infection.^1^–^7^

### Paronychia

Paronychia is a painful inflammatory reaction of the nail folds.^37^ It is difficult to treat and causes the nail folds to become sensitive to infection. Antiseptic creams and drying pastes have been reported to be useful to prevent infection of the nail fold.^6^,^7^ Fissures in the nails have also been treated by liquid bandages and glue.^1^–^36^

### Hand–foot skin reaction

In addition to practical measures to avoid friction, mild reactions have been treated successfully with urea or salicylic acid ointment. Xerosis cutis has also been managed with specifically formulated hand or foot emollients.^7^–^29^

### Mucosal disorders

There is little mention in the literature on the treatment of oral and nasal aphthae, (mucositis) and dry anal and vaginal mucosae. Symptomatic treatment available consists of oral gels, nasal and vaginal creams.^32^

### Alteration of patient quality of life

The pain and morbidity associated with targeted chemotherapy can be difficult for cancer patients to bear and have been shown to impact quality of life as well as interpersonal relationships.^9^,^11^ The use of cosmetics and appropriate skin-care management has shown objective improvements on quality of life. A pilot study found significantly improved self-image (*P* < 0.005 compared with baseline) on the Self-image Non melanoma skin cancer scale and anxiety (*P* < 0.01) on the STAI scale.^39^ An uncontrolled, monocentre study showed a decrease from baseline (*P* < 0.0003) on the Skindex questionnaire.^18^ A multicentre qualitative survey reported patient appreciation for beauty-care services for treating skin problems that helped patients cope better throughout the treatment period.^40^ Recent prospective studies have shown that proactive education on self-care behaviours significantly reduced anxiety (*P *= 0.032) on the VQ dermato-scale, contributing to better symptom management.^41^ Training seminars that teach both men and women appropriate skin care, camouflage and dressing techniques have been proposed to restore self-esteem, particularly for those patients with pre-existing low esteem.^42^–^43^

## Skin-care options

### Proactive treatment

Proactive treatment is critical as toxicity has been reported to arise as early as 2 days after the first treatment.^7^,^9^ There is no clear evidence which patients may be more susceptible.^7^ However, *Gallimont-Collen et al*. reported a correlation between both older age and atopic predisposition and higher incidence of xerosis.^32^ A retrospective survey of skin-toxicity management found that proactive intervention was warranted to obtain maximum benefit from EGFRI treatment and prevent dose change or interruption.^13^ The NCCN task force report also recommended initiating treatment even for mild reactions in case they become dose limiting.^1^ Early education and continued encouragement throughout treatment have been shown to benefit quality of life.^44^–^45^

### Skin cleansing

In the process of skin cleansing, dirt or cosmetics are removed along with the sebum associated with it, thus further drying damaged skin.^24^–^46^ This has been shown to be particularly detrimental to skin affected by chemotherapy where the skin barrier is already disturbed.^21^ Without professional guidance, patients tend to experiment with inappropriate self-care behaviours that aggravate the situation or irritate their sensitive skin.^18^–^45^ In the lack of evidence, authors recommend that patients avoid washing with soap.^7^–^31^ Recently, some authors have started producing helpful evidence. *Fluhr et al*. reported that combining an acidic cleanser and emollient (pH 5.5) improved barrier function, stratum corneum hydration and skin surface lipids.^21^ *Roé et al*. reported good control of secondary infections in a 30-patient, prospective, study of the management of cutaneous side-effects with the use of antiseptic soaps.^29^

### Skin hydration

Chemotherapy reduces the skin’s tolerance to cosmetic products.^21^ This distinctive reaction that cancer patients experience, has been attributed to an imbalance in the *stratum corneum* that ultimately results in a disruption of skin barrier function.^9^–^21^ General measures to prevent further deterioration of the barrier topical treatments should be continued, with care not to apply occlusive creams. The role of emollients is to protect the epidermal barrier.^9^ Topical application of moisturizers or emollients binds water with the *stratum corneum*, providing partial surface hydration. This has been shown repeatedly to improve epidermal barrier function and reduce the stinging, scaling, redness and cracks associated with chemotherapy-induced xerosis.^21^ Adequate hydration improves barrier function, reduces pruritus and prevents secondary infections due to scratching.^6^–^18^

Therefore, skin care with moisturizers, low-irritant cleansers and make-up is effective in improving skin hydration and controlling or covering up some cutaneous reactions.

### Photoprotection

*Lacouture* highlights observations that EGFRI toxicity often occurs in sun-exposed areas, which have later been further supported by clinical and experimental data.^47^–^48^ Daily photoprotection is important as the skin becomes more sensitive to UV radiation and in certain cases can lead to pigmentation changes.^49^–^50^ Symptom management and supportive care forums on dermatological-toxicity management recommend applying a broad-spectrum sunscreen [Sun protection factor(SPF) or UVA-PF, SPF 15 or higher] depending on the patient’s phototype and on the photosensitivity induced.^8^,^16^

### Deodorants

The use of antiperspirants or deodorants is a controversial topic as the effect of chemotherapy on the eccrine glands eliminates their need. However, the working group felt that in the interest of the patients’ well-being, deodorants and non-irritant perfumes may be used as part of maintaining a daily routine.

### Skin sensitivity

Individuals treated with EGFRIs often complain of having sensitive skin that stings, burns and itches, all of which may be due to cutaneous inflammation.^9^ Several authors recommend avoiding allergenic or irritant products such as alcohol, topical retinoid and benzoyl peroxide.^6^–^8^

### Dermatologist referral

Most symptoms either resolve spontaneously, or can be managed by the treating physician. However, dermatologist consultation is recommended when lesions are uncharacteristic, blistering, petechial or necrotic.^8^

## Discussion and recommendations

The association of cutaneous side-effects with the use of targeted chemotherapy is now well accepted. However, evidence-based support for the use of dermatological cosmetics as adjunctive therapy to manage these problems remains scarce. Practice is based on anecdotal reports, personal experience or studies with limited control, and currently, no standard recommendation on how to treat cutaneous side-effects of oncology treatments exists.

Most authors agree that skin care is an essential part of well-being and dermatologists should not deprive patients of this habit. The consistency of evidence and reported experience allowed the expert group making realistic suggestions on a treatment algorithm.

The literature analysed consistently supports the use of emollients and mild soaps, and a controlled study demonstrated significantly improved skin physiology and appearance with combined use of mild soap and emollients.^21^

To treat skin rash, the most common reaction reported to appear within the first 2 days of treatment, all authors unanimously recommended the use of non-occlusive emollients.

Sun exposure has been reported to worsen rash, hence the need for photo protection.^50^

The sensitive nature of the skin has often been described, leading numerous authors to suggest avoiding use of irritant products.^9^

Parenchyma and fissures were reported to be difficult to treat. Glue and liquid bandages as well as antiseptic creams were considered to be the most useful.

The use of antiperspirants is a controversial topic as the effect of chemotherapy on the eccrine glands eliminates their need. However, the working group felt that in the interest of patient well-being, deodorants and non-irritant perfumes may be used as part of maintaining a daily routine.

In terms of quality of life, studies showed significant improvements on anxiety and self-image when patients received adequate skin-care advice.^39^–^41^

To provide physicians with practical information, the following treatment algorithm was built from the available data and expert opinion. The algorithm proposes a baseline treatment followed by additional suggestions according to symptom severity.

The working group considers that all symptoms including folliculitis, xerosis, fissures, as well as hand and foot syndrome are linked to a skin barrier dysfunction. Maintaining skin barrier function using appropriate cosmetic products can control the severity of these symptoms. At the beginning of treatment, patients should receive information about dermatological skin-care products and education on appropriate use.^44^ This should be continued and encouraged throughout treatment.^51^ Symptoms should be evaluated all along the therapy and topical or systemic treatments may be added according to existing guidelines, if necessary. Dermatologist referral should be considered whenever symptoms worsen.

**Figure 1:**
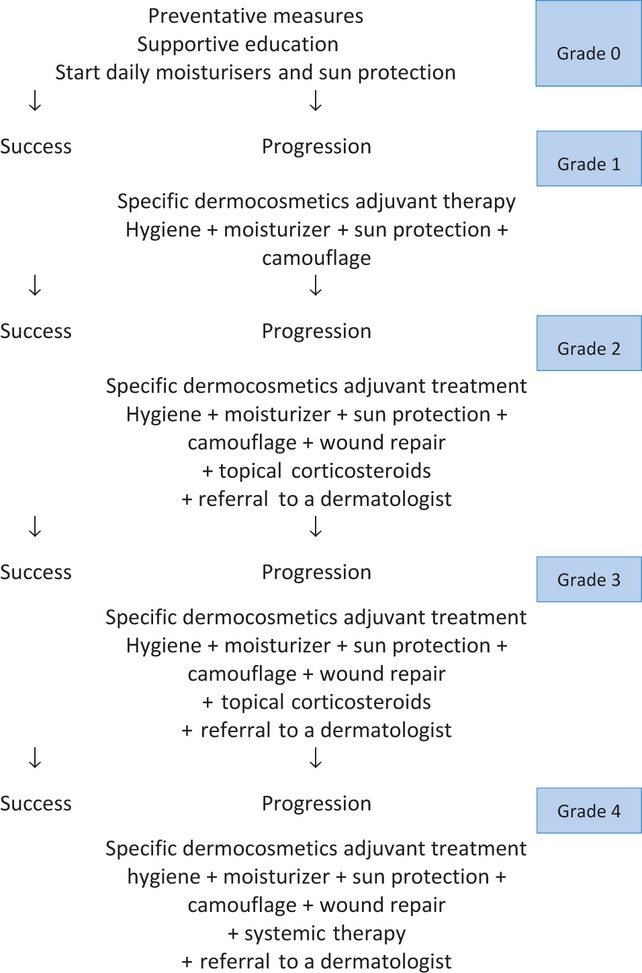
Proposed algorithm for the management of cutaneous toxicity associated with targeted therapies.

The following strategies are illustrated in the algorithm Fig. 1.

1. There is no evidence to suggest that skin cleansing should be avoided. Syndets with a pH of 5.5 are well tolerated and may be considered for use.
2. Daily application of a non-comedogenic moisturizing cream on both the face and body, irrespective of the chemotherapeutic agent prescribed, controls rash and xerosis. Consider oil-in-water vehicles for medical treatments and emollients containing humectants such as urea 5–10% or niacinamide.
3. Apply broad-spectrum sunscreen to the face and other exposed areas (i.e. neck and arms). SPF 15+/UVA-PF level according to phototype or expected photosensitivity
4. Well-being was improved by covering disfiguring erythema and pallor with non-comedogenic make-up. Avoid occlusive make-up if folliculitis is severe.
5. Fruit acids, antibacterials or benzoyl peroxide are not helpful to treat rash. Furthermore, they may cause irritation and be harmful.
6. Antiseptics and wound-healing creams maybe helpful in managing fissures and parenchyma.

The authors recognize that no systematic review was performed on available literature and hence relevant studies may not be cited. However, the authors feel that with this recommendation, a first attempt was made to provide guidance to the physicians who are dealing daily with skin-care problems in patients undergoing chemotherapy.

## Conclusion

The present guidelines are intended to support optimization of therapeutic management of cutaneous side-effects and to improve the quality of life of oncology patients.

However, the authors recognize that further research is needed to test skin-care products in this population suffering from particularly sensitive skin.
